# Global methylation silencing of clustered *proto-cadherin* genes in cervical cancer: serving as diagnostic markers comparable to HPV

**DOI:** 10.1002/cam4.335

**Published:** 2014-11-21

**Authors:** Kai-Hung Wang, Cuei-Jyuan Lin, Chou-Jen Liu, Dai-Wei Liu, Rui-Lan Huang, Dah-Ching Ding, Ching-Feng Weng, Tang-Yuan Chu

**Affiliations:** 1 Department of Research, Center for Cervical Cancer Prevention, Buddhist Tzu Chi General HospitalHualien, Taiwan; 2 Institute of Medical Sciences, School of Medicine, Tzu Chi UniversityHualien, Taiwan; 3 Department of Life Science and Institute of Biotechnology, National Dong Hwa UniversityHualien, Taiwan; 4 Department of Radiation Oncology, Buddhist Tzu Chi General HospitalHualien, Taiwan; 5 Department of Obstetrics and Gynecology, Shuang Ho Hospital, Taipei Medical UniversityTaipei, Taiwan; 6 Department of Obstetrics and Gynecology, Buddhist Tzu Chi General HospitalHualien, Taiwan

**Keywords:** Cancer biomarker, cervical cancer, clustered *proto-cadherin*, DNA methylation, HPV

## Abstract

Epigenetic remodeling of cell adhesion genes is a common phenomenon in cancer invasion. This study aims to investigate global methylation of cell adhesion genes in cervical carcinogenesis and to apply them in early detection of cancer from cervical scraping. Genome-wide methylation array was performed on an investigation cohort, including 16 cervical intraepithelial neoplasia 3 (CIN3) and 20 cervical cancers (CA) versus 12 each of normal, inflammation and CIN1 as controls. Twelve members of clustered *proto-cadherin* (*PCDH*) genes were collectively methylated and silenced, which were validated in cancer cells of the cervix, endometrium, liver, head and neck, breast, and lung. In an independent cohort including 107 controls, 66 CIN1, 85 CIN2/3, and 38 CA, methylated *PCDHA4* and *PCDHA13* were detected in 2.8%, 24.2%, 52.9%, and 84.2% (*P* < 10^−25^), and 2.8%, 24.2%, 50.6%, and 94.7% (*P* < 10^−29^), respectively. In diagnosis of CIN2 or more severe lesion of the cervix, a combination test of methylated *PCDHA4* or *PCDHA13* from cervical scraping had a sensitivity, specificity, positive predictive value, and negative predictive value of 74.8%, 80.3%, 73%, and 81.8%, respectively. Testing of this combination from cervical scraping is equally sensitive but more specific than human papillomavirus (HPV) test in diagnosis of CIN2 or more severe lesions. The study disclosed a collective methylation of *PCDH* genes in cancer of cervix and other sites. At least two of them can be promising diagnostic markers for cervical cancer noninferior to HPV.

## Introduction

Discovery and application of cancer biomarkers has been hampered by the extensive heterogeneities of tumorigenesis. While tumor-promoting genetic and epigenetic changes may differ among cancers of various sites and individual cancers of the same site 1, the universal carcinogenic effect of human papillomavirus virus (HPV) in virtually all cervical cancers stands out as an exception 2. The invariable site of transformation at the squamous-columnar (SC) junction 3 allows a precise target for screening. Various diagnostic biomarkers such as high-risk HPV DNA, p16, and methylated genes have been discovered from scraping specimens at this site with unprecedented success 4–7.

In the decade-long evolution of carcinoma in situ (CIS) toward invasion, extensive epigenetic changes are required for epithelial to mesenchymal transition (EMT) 8 before invasion and metastasis. This involves methylation silencing of genes functioned in cell adhesion 9. Among the superfamilies of cell adhesion molecules, clustered *proto-cadherin* (*PCDH*) is the largest one. The three clusters, namely *α*,*β*, and *γ PCDH* gene clusters (Human Genome Organization nomenclature *PCDHA@*,*PCDHB@*, and *PCDHG@*, respectively), distribute in tandem at chromosome 5q31. *PCDHA@* and *PCDHG@* contain a tandem array of 15 and 22 variable exons, respectively, and each is controlled by a specific promoter. Expression of these genes involves both differential promoter activation and alternative pre-mRNA splicing to the downstream constant exon 10,11.

Taking cervical carcinogenesis as a model, this study aims to globally search for methylated genes that are specific for cancer invasion. Among the overrepresented genes were members of clustered *PCDH* genes, which were silence by methylation in cancer cell lines of multiple sites including the cervix. Progressive methylation of two flanking members of *PCDHA@, PCDHA4*, and *PCDHA13* was observed in an independent cohort. In comparison with HPV test, methylation test of the two genes is more specific and equally sensitive in detecting in CIN2 or more severe lesions.

## Materials and Methods

### Studied subjects

This study was approved by Institutional Review Board of Tzu Chi General Hospital, Hualien, Taiwan, and informed consent was signed by each subject. Subjects including an investigation cohort and a testing cohort were enrolled from December 2005 to May 2013. In the investigation cohort, thirty six each of cases and controls were enrolled. The case group included cervical scrapings of 16 squamous cell carcinomas (SCC), 4 adenocarcinomas (AdenoCA), 14 CIS (carcinoma in situ), and 2 cervical intraepithelial neoplasia 3 (CIN3). The control group included 12 scrapings each of cytology/histology diagnosis of “negative for cervical neoplasia” (Normal), “reactive changes or inflammation” (Inflammation), and CIN1. The testing cohort was consisted of 107 Normal or Inflammation, 66 CIN1, 85 CIN2/3, and 38 invasive carcinomas (CA). Demographic data of these two cohorts were showed in Table1. In both cohorts, subjects with newly diagnosed cervical neoplasia were enrolled and managed according to a standard guideline issued by the National Health Research Institute of Taiwan (http://tcog.nhri.org.tw/doc/gog_a.pdf). All subjects with CIN1 or more severe lesions were examined by colposcopy with a biopsy tissue proof. Healthy women undergoing regular Pap smears with a “Normal” or “Inflammation” results were recruited as controls.

**Table 1:** Demorgraphic data of the investigation and testing cohorts

						CA					
	Normal	Inflammation	CIN1	CIN2	CIN3	Total	Stage 1	Stage 2	Stage 3	Stage 4	Total
Investigation cohort											
Number of case	12	12	12	0	16	8	4	4	4	20	72
Age ± SE	57.1 ± 2.2	48.1 ± 2.4	43.8 ± 3.6	Nil	49.2 ± 3.7					56.1 ± 3.1	51.3 ± 1.5
Testing cohort											
Number of case	107		66	19	66	11	18	4	5	38	296
Age ± SE	50.2 ± 1.1		43.6 ± 1.5	46.1 ± 4.0	55.4 ± 1.9					57.9 ± 2.4	50.6 ± 0.8

CIN3, cervical intraepithelial neoplasia 3.

### Clinical specimens and nucleic acid extraction

Cervical tissues with CIN or invasive carcinoma were procured during the procedures of colposcopic biopsy, conization, and radical hysterectomy. Normal cervical tissues were obtained from patients receiving hysterectomy for benign tumors of the uterus. All the tissue specimens were collected within 20 min after resection and were frozen in −80°C. Cervical scrapings were collected by a cytobrush before colposcopy, cervical biopsy, or other procedures. The scrapings were transferred into a universal tube containing 3 mL of phosphate buffered saline. After thorough agitation, dispensing, they were stored at −80°C. Nucleic acids were extracted from cervical scrapings and the tissues by using DNA Mini Kit (Qiagen, Hilden, Germany) according to the manufacturer's instructions.

### DNA methylation array and data analysis

DNA extracted from cervical scrapings of the case and control groups were each pooled and fragmented by sonication. Methylated DNA fragments were enriched by immuneprecipitation using the MeDIP kit (Diagenode, Sparta, NJ), amplified by using a whole genome amplification kit (Sigma, St. Louis, MO) and examined on the NimbleGen DNA Methylation Array (Roche, Boulder, CO) analysis. For array hybridization, the MeDIP DNA of cases and controls were differentially labeled with Cy5 and Cy3 and cohybridized on an array containing 385,000 probes which recognize CpG islands and CpG sites adjacent (at −250 to 500 bp) to transcription start sites genome-wide. From the scaled log2-ratio data of each probe, a fixed-length (750 bp) window was placed around each consecutive probe and the one-sided Kolmogorov–Smirnov (KS) test was applied to determine whether probes were drawn from a significantly more positive distribution of intensity log-ratios than those in the rest of the array. The resulting score (peak score) for each probe was the −log10 *P*-value from the windowed KS test around that probe.

### Cell culture and 5′-aza- 2′-deoxycytidine treatment

In total, 17 cell lines derived from cancer of the cervix (SiHa, Caski, C33A, and HeLa), breast (MCF7, MDA-MB-231, and MDA-MB-435), lung (CL1-0 and A549), liver (SK-Hep1, HuH6, and Hep3B), head and neck (A253, FaDu, and Detroit 562), and endometrium (RL95-2 and HTB-111) were used for validation. The HeLa, MCF7, MDA-MB-231, and MDA-MB-435 cells were cultured with Dulbecco's modified Eagle's medium; the C33A, A253, FaDu, and Detroit 562 cells were cultured with minimum essential medium; and the other cells were cultured in Rosewell Park Memorial Institute-1640 medium. All mediums contained 10% fetal bovine serum and 1% penicillin/streptomycin. For demethylation treatment, 1 × 10^6^ cells were seeded in medium containing 5′-aza-2′-deoxycytidine (5-aza) (Sigma) and the medium was replaced every day for a total of 4 days. Different concentrations of 5-aza (2.5, 5, 7.5, and 10 *μ*mol/L) were tried in pilot studies for each cancer cell. The 10 *μ*mol/L concentration turned out to give the best demethylation effect and did not affect cell survival.

### RT-PCR analysis of clustered *PCDH* transcripts

Total RNA was extracted using TRIzol reagent (Invitrogen, Carlsbad, CA) and was reverse transcribed using the First Strand cDNA Synthesis kit (Roche) and subjected to PCR with primers listed in Table S1. The following PCR condition was used with minor, uncritical variations was designed for each *PCDH* gene that gave amplification at the logarithm phase: one cycle at 95°C for 10 min, 35 cycles at 95°C for 30 sec/58–62°C for 30 sec/72°C for 30 sec, and one cycle at 72°C for 5 min. As the loading control, the *β-actin* gene was amplified under the same condition. The relative expression level was normalized by *β-actin* and the ratio of the density of PCR products in the agarose gel was calculated by using the ImageJ program (National Institutes of Health, Rockville Pike, Maryland).

### Bisulfite genomic sequencing

Genomic DNA was bisulfite-modified using the DNA Methylation Gold Kit (Zymo Research). Based on the methylation array results, we focused on the hypermethylation region of each gene. The primers were designed from the MethPrimer website (http://www.urogene.org//methprimer/index1.html) (Table S1) and annealed to bisulfite DNA at 55°C. Amplified products were cloned into TA-cloning plasmids, transformed into competent cells, and the sequences were determined from randomly chosen colonies.

### Methylation-specific PCR (MS-PCR)

Based on the BGS results, primers covering the highly distinctive methylation sites between normal and cancer specimens were designed for methylation-specific PCR (MS-PCR) via the MethPrimer website. MS-PCR was done by using Gold Taq DNA polymerase (Applied Biosystems, Foster City, CA). Table S1 lists the primers used. DNA of peripheral white blood cells was used for nonmethylated template control and those treated with CpG methyltranferase M. SssI (New England Biolab, Ipswich, MA) as the methylated control. The relative ratio of density of amplified product from methylation-specific PCR (MSP) and unmethylation-specific PCR (USP) resolved in the agarose gel was calculated by using the ImageJ program.

### HPV detection and typing

The presence of HPV DNA and genotypes was determined by consensus PCR and reverse blot hybridization as described previously 12. Briefly, the MY11 and biotinylated GP6+ consensus primers were used to amplify a fragment of ∼192 bp in the L1 open reading frame of the HPV genome for 40 cycles. The PCR products were then hybridized with an Easychip HPV Blot (King Car; Yuan-San, I-Lan County, Taiwan), which included oligonucleotide probes of 39 different HPV types, and were visualized with biotinylated antibodies and alkaline phosphatase conjugation.

### Statistical analysis

Data was analyzed by using SPSS 15.0 (SPSS, Inc., Chicago, IL). The relationship between cervical neoplasia and biomarkers was analyzed by Fisher's exact test.

## Results

### Widespread methylation of clustered *PCDH* genes in cervical cancers

**Figure 1:**
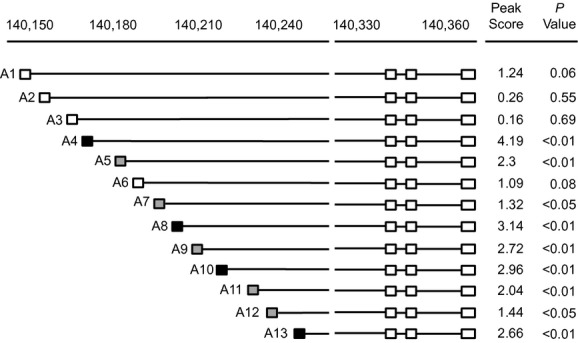
Methylation of members of the *PCDHA@* gene cluster in cervical cancer. Genomic organization of the 13 members of the *PCDHA@* gene cluster reveals a gene-specific exon 1 followed by shared exon 2, 3, and 4 (boxes). Peak score and *P*-value of the most significant peak of each gene are given. The first exons with a most significant peak scores higher than 1.3 (*P *<* *0.05) are indicated by gray boxes, and those with an average peak score higher than 1.3 are indicated by black boxes.

In total, 2152 peaks representing 2227 genes were found to be specifically methylated in the cancer/precancer group with an “average peak score” over 1.3 (or *P *<* *0.05) (Table2 and Data S1). Gene ontology analysis for biological process revealed enrichment of genes involved in cell adhesion signaling (*n* = 67) and cell adhesions (*n* = 78) with Enriched Score of 6.94 and 2.98, respectively (Table2). A lot of these cell adhesion genes are members of clustered *PCDH* genes. They include four members in each of the *PCDHA@* (*PCDHA4*,*A8*,*A10*,*A13*), *PCDHB@* (*PCDHB3*,*B6*,*B7*,*B14*), and *PCDHG@* (*PCDHGA12*,*GB6*,*GB7*,*GC3*) cluster (Table3). In particular, individual *PCDH* genes in clusters are hypermethylated consecutively. For instance, at a less stringent criterion of “any peak score” of >1.3 (or *P *<* *0.05), nine consecutive members (from *PCDHA4* to *PCDHA13*) of the *PCDHA@* were hypermethylated in the case group but not the control group (Fig.1). These data suggest that clustered *PCDH* genes may be hypermethylated in cervical cancer/precancer lesion.

**Table 2:** Summary and functional annotation of hypermethylated genes overrepresented in cervical cancer scraping

Biological process ID	Term	Number of hypermethylated gene	*P*-value^1^	FDR	Enriched score (−log10 *P*-value)
BP00274	Cell communication	174	6.13E-11	7.73E-08	10.21
BP00102	Signal transduction	400	3.25E-10	4.10E-07	9.49
BP00120	Cell adhesion-mediated signaling	67	1.14E-07	1.43E-04	6.94
BP00122	Ligand-mediated signaling	58	0.0006	0.77	3.21
BP00124	Cell adhesion	78	0.0010	1.30	2.98
BP00193	Developmental processes	230	0.0014	1.77	2.85
BP00141	Transport	143	0.0051	6.28	2.29
BP00004	Carbohydrate transport	12	0.0057	7.00	2.24

FDR, false discovery rate.

1 Modified Fisher exact *P*-value identified by DAVID.

**Table 3:** Partial list of cell adhesion genes found to be enriched in the cancer/precancer group in methylation array study

Peak ID	Locus	No. of peak^1^ with score >1.3	Average peak score	Score of the most significant peak	Feature^2^-to-peak distance	Gene name
106	5q31	6	3.05	4.19	460	PCDHA4
379	5q31	6	2.35	3.14	381	PCDHA8
568	5q31	14	2.14	2.96	−64	PCDHA10
784	5q31	14	1.99	2.66	−113	PCDHA13
1846	5q31	3	1.52	1.56	−135	PCDHB3
941	5q31	2	1.9	2.18	79	PCDHB6
1177	5q31	4	1.79	2.30	−59	PCDHB7
518	5q31	4	2.19	3.22	−12	PCDHB14
878	5q31	5	1.93	2.71	208	PCDHGA12
1872	5q31	2	1.51	1.62	−514	PCDHGB6
144	5q31	7	2.88	4.02	234	PCDHGB7
551	5q31	9	2.16	2.92	325	PCDHGC3
387	7q31	7	2.34	3.19	−519	NRCAM
661	10q23.1	2	2.06	2.15	−11	PCDH21
1283	11q23	2	1.74	1.74	−47	DSCAML1
773	11q24.1	5	1.99	2.34	−356	ASAM
982	13q21.1	4	1.87	2.79	−260	PCDH17
1563	16q22.1	3	1.63	1.85	−257	CDH5
471	16q22.1	9	2.23	2.95	−185	CDH16
985	16q24.3	5	1.87	2.17	−560	CDH15
1834	19p13.2	11	1.52	1.86	−74	ICAM5
1767	19q13.2	2	1.55	1.62	115	CEACAM8
640	19q13.2	5	2.08	2.31	−566	CEACAM16
1920	20q13.1	6	1.48	1.96	−295	CDH22
619	Xq28	4	2.1	2.66	−483	L1CAM

1 Peaks are detected (from the *P*-value data) from at least two probes that have *P* < 0.05 which is equal to a −log(10) value or peak score of 1.3.

2 The feature is the transcription start site.

### Clustered *PCDH* genes are methylation-silenced in multiple carcinoma cells

**Figure 2:**
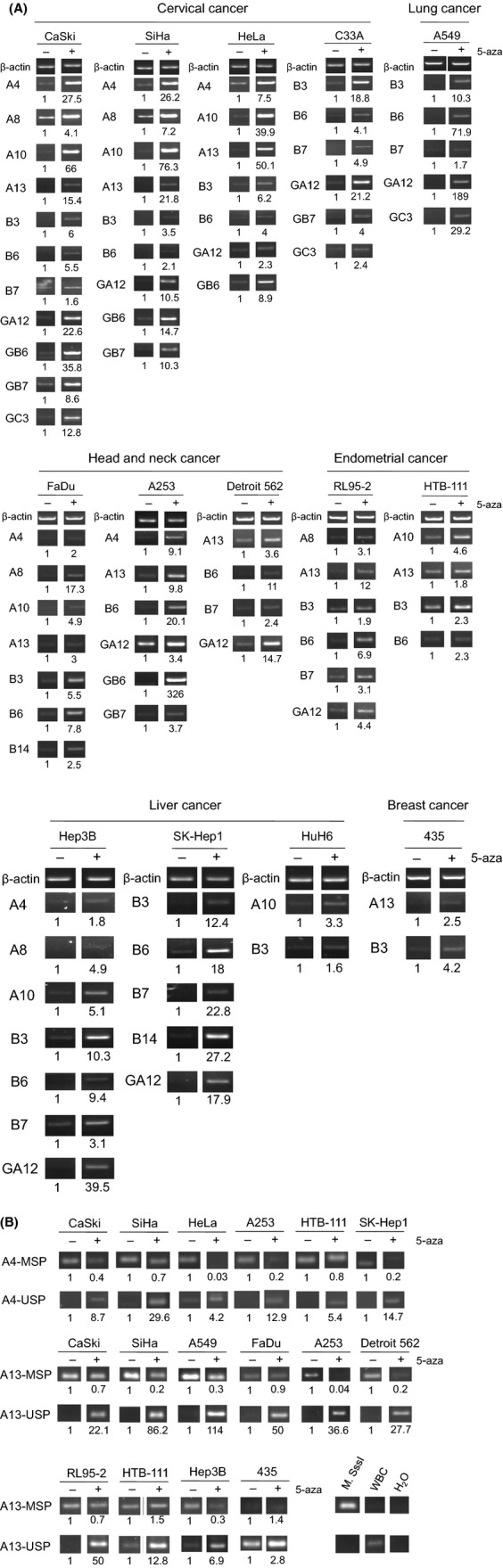
Methylation silence of members of clustered *PCDH* genes in cancer cells. (A) mRNA expression of clustered *PCDH* genes were analyzed by RT-PCR in cancer cells with (+) or without (−) demethylation treatment with optimal doses of 5-aza. The ratio of the density of PCR product in agarose gel was calculated by using ImageJ program and listed under the gel band. Only those showing low-expression deserving demethylation treatment were showed. (B) Demethylation of *PCDHA4* and *PCDHA13* after 5-aza treatment was confirmed by methylation-specific (MSP) and nonmethylation-specific (USP) PCR. The M. SssI methylase-treated WBC DNA was used as a methylation control (M. SssI) and untreated DNA was used as nonmethylated control (WBC). Non-DNA blank control (H_2_O) was used as a negative control for PCR reaction. The relative ratio of MSP and USP density was calculated by using ImageJ program and listed under the PCR product. PCR, polymerase chain reaction.

**Figure 3:**
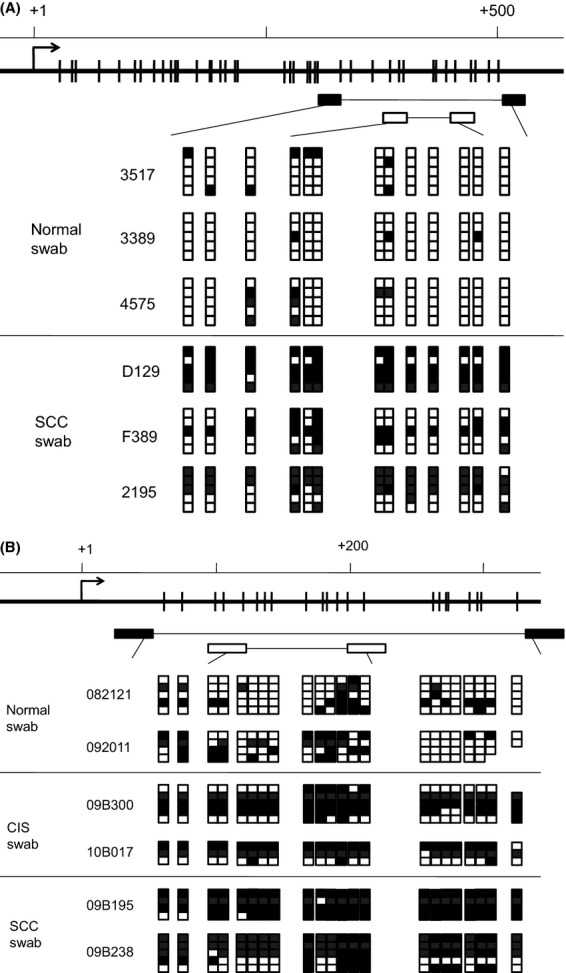
Hypermethylation of the CpG island at exon 1 of the *PCDHA4* and *PCDHA13* genes in cervical cancers and normal controls. Genomic structure around the first exon of the *PCDHA4* (A) and *PCDHA13* (B) genes are demonstrated in the upper panel. The CpG sites around the first exon of the two genes were marked as vertical bars and the transcription start site as arrow at “+1”. Primers designed for bisulfite genomic sequencing (BGS) are indicated as black boxes and for MSP are indicated as empty boxes under the CpG sites. The lower panel shows the BGS results of samples of SCC, CIS, and Normal. Methylated and nonmethylated CpG sites are marked by closed and open squares, respectively. SSS, squamous cell carcinomas; CIS, carcinoma in situ.

The twelve methylated members of clustered *PCDH* were underexpressed in a wide variety of cancer cell lines including carcinoma of the cervix (4/4), endometrium (2/2), liver (3/3), head and neck (3/3), breast (1/3), and lung (1/2). A majority of these underexpressed genes could be reexpressed upon treating the cells with 10 *μ*mol/L 5-aza, a demethylation agent (Table4, Fig.2A). To further exploration of this *PCDHA@* cluster, we chose the two flanking members, *PCDHA4* and *PCHA13*, for further characterization. The methylation status of these two genes, before and after demethylation treatment, was confirmed by MS-PCR, in which methylation product was reduced and unmethylation one was increased after demethylation treatment (Fig.2B). Detailed mappings of methylation at CpG islands around the first exon were shown in Figure3. All the CpG sites, including five each recognized by the MS-PCR primers for *PCDHA4* and *PCDHA13*, were hypermethylated in the SCC or CIS samples, whereas low methylation densities were noted in the normal samples. These results suggest most of clustered *PCDH* genes, especially *PCDHA4* and *PCDHA13*, were methylation-silenced, not only in cervical cancer cells but also in other cancer cells.

**Table 4:** Methylation silencing of *PCDH* genes in cancer cells of the cervix, liver, head and neck (H&N), endometrium, lung, and breast

		Cervix				Liver			H & N			Endometrium		Lung		Breast		
Gene name	Average peak score	CaSki	SiHa	HeLa	C33A	Hep3B	SK-Hep1	HuH6	A253	FaDu	Detroit 562	RL95-2	HTB-111	A549	CL1-0	MB 435	MB 231	MCF7
PCDHA4	3.05	+/+	+/+	+/+	−/−	+/+	+/−	+/−	+/+	+/+	−/−	−/−	+/−	−/−	−/−	+/−	+/−	−/−
PCDHA8	2.35	+/+	+/+	−/−	−/−	+/+	+/−	+/−	−/−	+/+	−/−	+/+	−/−	−/−	−/−	−/−	−/−	−/−
PCDHA10	2.14	+/+	+/+	+/+	−/−	+/+	+/+	+/+	−/−	+/+	−/−	−/−	+/+	−/−	−/−	−/−	−/−	−/−
PCDHA13	1.99	+/+	+/+	+/+	−/−	+/−	+/−	+/−	+/+	+/+	+/+	+/+	+/+	−/−	−/−	+/+	+/−	−/−
PCDHB3	1.52	+/+	+/+	+/+	+/+	+/+	+/+	+/+	−/−	+/+	+/−	+/+	+/+	+/+	−/−	+/+	−/−	−/−
PCDHB6	1.9	+/+	+/+	+/+	+/+	+/+	+/+	+/−	+/+	+/+	+/+	+/+	+/+	+/+	−/−	+/−	−/−	−/−
PCDHB7	1.79	+/+	−/−	−/−	+/+	+/+	+/+	+/−	−/−	+/−	+/+	+/+	−/−	+/+	−/−	−/−	+/−	−/−
PCDHB14	2.19	−/−	−/−	−/−	−/−	−/−	+/+	−/−	−/−	+/+	−/−	−/−	−/−	−/−	−/−	−/−	−/−	−/−
PCDHGA12	1.93	+/+	+/+	+/+	+/+	+/+	+/+	+/−	+/+	+/−	+/+	+/+	+/−	+/+	−/−	−/−	−/−	−/−
PCDHGB6	1.51	+/+	+/+	+/+	−/−	+/−	+/−	+/−	+/+	+/−	−/−	−/−	−/−	−/−	−/−	+/−	−/−	−/−
PCDHGB7	2.88	+/+	+/+	−/−	+/+	−/−	−/−	−/−	+/+	+/−	+/−	+/−	−/−	−/−	−/−	+/−	−/−	−/−
PCDHGC3	2.16	+/+	+/−	−/−	+/+	−/−	−/−	−/−	−/−	+/−	−/−	−/−	+/−	+/+	−/−	+/−	−/−	−/−

+/+: low expression and re-expressed after demethylation, +/−: low expression and not reexpressed after demtehylation, −/−: normal expression and not reexpressed after demthylation. +/+ was highlighted in dark grey shade and +/− in light grey shade.

### Methylation of the *PCDHA4* and *PCDHA13* genes are strongly correlated with severity of cervical neoplasia

In an independent testing cohort of cervical scrapings of different severity, progressive methylation of the *PCDHA4* was noted from 2.8% in Normal or Inflammation, 24.2% in CIN1, 52.9% in CIN2/3, to 84.2% in CA (*P* < 10^−25^). For *PCDHA13*, methylation was noted from 2.8%, 24.2%, 50.6%, to 94.7%, respectively (*P* < 10^−29^). As a comparison, high-risk HPV was detected in 77.6% of CIN2/3 and 86.8% of CA, but was also found in 15.9% of Normal or Inflammation and 45.5% of CIN1 (*P* < 10^−21^, Table5). Methylation of these two genes was further verified in tissue specimens in this cohort. As showed in Table6, methylation of either of the two genes was noted in 12.5% of Normal cervix, 11.1% of CIN1, 57.1% of CIN2/3, 78.9% of invasive CA and 100% of metastatic CA (*P* < 10^−6^). The data demonstrate a positive correlation of frequency of methylation of *PCDHA4* and *PCDHA13* with severity of cervical neoplasia, and suggest a potential to be applied in clinical diagnosis.

**Table 5:** Methylation of *PCDHA4* and *PCDHA13*, and high-risk HPV in the testing cohort of cervical scraping

Pathology	No.	Age ± SE	*PCDHA4*^me^	%	*PCDHA13*^me^	%	*A4*^me^ or *A13*^me^	%	High-risk HPV	%
Normal	107	50.2 ± 1.1	3/107	2.8	3/107	2.8	6/107	5.6	17/107	15.9
CIN1	66	43.6 ± 1.5	16/66	24.2	16/66	24.2	28/66	42.4	30/66	45.5
CIN2/3	85	53.3 ± 1.7	45/85	52.9	43/85	50.6	55/85	64.7	66/85	77.6
CA	38	58.0 ± 2.2	32/38	84.2	36/38	94.7	37/38	97.4	33/38	86.8
*P*-value^1^			2.8 × 10^−26^		1.5 × 10^−30^		1.2 × 10^−31^		1.1 × 10^−22^	

*PCDHA4*^me^: methylation of *PCDHA4, PCDHA13*^me^: methylation of *PCDHA13*. HPV, human papillomavirus; CIN3, cervical intraepithelial neoplasia 3.

1 Fisher's exact test.

**Table 6:** Methylation of *PCDHA4* and *PCDHA13* and high-risk HPV in cervical tissues

Pathology	Age ± SE	*PCDHA4*^me^	%	*PCDHA13*^me^	%	*PCDHA4*^me^ or *PCDHA13*^me^	%
Normal	46.0 ± 2.5	2/14	14.3	1/16	6.3	2/16	12.5
CIN1	38.0 ± 4.2	0/7	0.0	1/9	11.1	1/9	11.1
CIN2/3	46.1 ± 3.4	6/13	46.2	6/13	46.2	8/14	57.1
CA	55.4 ± 2.1	26/37	70.3	23/33	69.7	30/38	78.9
Metastatic CA	59.4 ± 6.3	6/6	100.0	6/7	85.7	7/7	100.0
*P*-value^1^		*P *=* *5.6 × 10^−6^		*P *=* *5.7 × 10^−6^		*P *=* *1.3 × 10^−7^	

CIN3, cervical intraepithelial neoplasia 3.

1 Fisher's exact test.

### Testing of methylated *PCDHA4* or *PCDHA13* is more specific than HPV test in detecting CIS and invasive cervical cancer

We analyzed the performance of testing methylated *PCDHA* and high-risk HPV in detection of cervical neoplasia of different severity in the testing cohort. Both methylated *PCDHA* tests had a lower sensitivity but higher specificity than HPV test in detecting CIN2+, CIN3+ or CA. A combination test of methylation of *PCDHA4* or *PCDHA13* achieved a better sensitivity (97.4%) and specificity (65.5%) than the HPV test in detecting CA. For early detection of CIN2 lesion, testing of methylated *PCDHA4* or *PCDHA13* is equally sensitive (74.8%) but more specific (80.3%) than HPV test (Table7).

**Table 7:** Performance of methylation of *PCDHA4* or *A13* in comparison with high-risk HPV test in the testing cohort of cervical neoplasia

	*PCDHA4*^me^				*PCDHA13*^me^				*PCDHA4*^me^ or *PCDHA13*^me^				High-risk HPV			
Detection target	Sen	Spe	PPV	NPV	Sen	Spe	PPV	NPV	Sen	Spe	PPV	NPV	Sen	Spe	PPV	NPV
CIN2+	62.6	89.0	80.2	77.0	64.2	89.0	80.6	77.8	74.8	80.3	73.0	81.8	75.6	72.8	66.4	80.8
CIN3+	66.3	85.9	71.9	82.5	71.2	87.5	75.5	84.8	79.8	77.6	65.9	87.6	75.0	67.7	55.7	83.3
CA+	84.2	75.2	33.3	97.0	94.7	76.0	36.7	99.0	97.4	65.5	29.4	99.4	86.8	58.5	23.6	96.8

Sen, sensitivity; Spe, specificity, PPV, positive predictive value; NPV, negative predictive value; HPV, human papillomavirus; CIN3, cervical intraepithelial neoplasia 3.

Given that HPV type 16 and 18 are the most oncogenic HPV types causing over 70% of cervical cancer worldwide and may serve as a specific diagnostic marker 13, we tested the performance of combination of HPV16/18 with the two DNA methylation markers. As showed in Table S2, testing for the presence of any of these four markers in cervical scrapings increased the sensitivity but decreased the specificity for detecting CIN2 or more severe lesions. The effect is more prominent in detection of CIN2 and CIN3 than of CA. A benefit of increasing sensitivity with less compromise of specificity was found when HPV16/18 was added to methylated *PCDHA4*, but not to the methylated *PCDHA13* or the combination of two *PCDHA@* members.

## Discussion

In this genome-wide promoter methylation study, collective methylation silencing of clustered *PCDH* genes was observed in cervical cancers. In addition, carcinoma cells of different origins including the endometrium, the head and neck, the liver, the lung, and the breast were each found to harbor silenced *PCDH* genes of differing spectra. This finding is consistent with the long range epigenetic silencing (LRES) of clustered gene loci in cancers, as illustrated in breast cancer 14 and in Wilm's tumor 15. Recently, the cis-regulatory elements of *PCDHA@* were identified and the possible mechanism of competition between individual variable exon promoters was characterized 16. Interestingly, as shown in Table4, not all of the silenced members within the *PCDH* locus can be reexpressed by demethylation treatment. This is in accordance with the findings of coordinate suppression of a 4-Mb segment in colon cancer where silencing of individual genes within a LRES locus appears to be dependent on a domain-wide nonpermissive chromatin configuration rather than the methylation status 17.

Differing from previous genome-wide studies of DNA methylation in cancers, which used cancer tissues as sources of exploration 18–20, this study discovered methylated markers from swab samples of the spectrum of cervical carcinogenesis. Tumor tissues, especially those from precancerous lesions and the normal epithelial tissue, are comprised mainly of stromal cells and typically do not represent the neoplastic or epithelial cells. In contrast, cervical scrapings are comprised mainly of epithelial cells with a minor population of inflammatory cells, and can be a better source to search for biomarkers. A design of pooling samples of normal, inflammatory, and mildly dysplastic scrapings may reduce the individual variations of cell components and more specifically target on severe neoplasia. Indeed, candidate genes were validated in a high yield. Eleven of the 12 candidate clustered *PCDH* genes were confirmed in cervical neoplasia and most of them were also methylation-silenced in other cancer cells tested (Table4).

In accordance with a general phenomenon of EMT with loss of cell–cell adhesions during the invasion of cancer, numerous cell adhesion genes have been reported as silenced by DNA methylation in carcinomas. These include classical cell adhesion genes such as *CDH1* (*E-cadherin*) 21, *CDH4* (*R-cadherin*) 22, and *CDH13* (*T-cadherin*) 23, and nonclustered *PCDH* genes such as *PCDH10* 24,25 *PCDH20* 26, *FAT4* 27. Methylations of clustered *PCDH* genes are much less reported previously 15,28. The present study showed a widespread methylation silencing of clustered *PCDH* genes in multiple cancer cells. The finding that *PCDHA4* and *PCDHA13* are methylated drastically in CIN2/3 and invasive CA stages suggests a role of these clustered adhesive molecules in maintaining the integrity of normal and dysplastic cells.

Clustered *PCDH* genes are predominantly expressed in the brain and are sophisticatedly expressed during development of central nerve system 10. A complex system of gene regulation, expression, protein cleavage, and interaction among different members was observed 10,11. Studies of functions of these clustered cell adhesion molecules are complicated by their molecular diversity with cis-homodimers and cis-heterodimers on the same cell surface and by cross-regulations of the encoding genes 29,30. Recently, the mosaic or mixed DNA methylation states of *PCDHA*@ genes have been characterized in mouse neural cells and in vivo 31. This individual cell-specific and allele-specific control of these clustered genes may be responsible for development of cell identities of complex and highly organized tissues. Echoing to this notion, this study revealed down-regulation of specific spectra of clustered *PCDH* genes in cancer cells of differing sites, suggesting tissue-specific adhesion profiles that are overridden in cancer invasion.

Each one or specific combinations of hyper-methylated genes unveiled in this study may be effective biomarkers of cancers. As shown in this study, the frequency of methylation of *PCDHA4* and *PCDHA13* correlates well with the progression of cervical carcinogenesis. Comparing to high-risk HPV test, methylated *PCDHA4* and *PCDHA13* were as frequently found in invasive cervical cancer but was much less frequently found in normal or CIN1 (Table5). In detection of CIN2/3 or more severe lesions, a combination test of methylation of either of the two genes achieved a better specificity (80.3% vs. 72.8%) and equal sensitivity (74.8% vs. 75.6%) as compared with the HPV test. In detection of CIN3 or more severe lesions, it even surpasses the HPV test (specificity 77.6% vs. 67.7%; sensitivity 79.8% vs. 75.0%). Currently, the main concern of using HPV test as screening and triage tool for high-grade cervical lesions is its lower specificity, due to the fact that most HPV infections are transient and only the persistent infection is associated with an increased risk of high-grade CIN and cancer. While specifically testing HPV type 16 and 18 may be more specific and adding HPV16/18 to the methylated gene markers has been showed to improve the sensitivity of detecting HSIL or above lesions 32, our data showed an improvement of performance when it was combined with *PCDHA4* methylation marker, but not for *PCDHA13*, which performed better than *PCDHA4*, or the combination of the two *PCDHA*s. Our data reveal the combination test of these two methylated genes can be a promising test in early detection of cervical cancer, which is at least as good as HPV test and even better when specifying the diagnostic target to mare severe lesions of CIS or CA (Table7).

Dozens of methylated genes have been discovered to aid the screening and diagnosis of cervical cancer 33. Among them, *CADM1*,*MAL*, and *PCDH10* have been established as tumor suppressor genes, and their methylation are highly related to the severity of cervical noeplasia 33–35. They have been investigated as triage tool for equivocal Pap smear result (*PCDH10*) 34, or for high-risk HPV infection (*CADM1* and *MAL*) 35,36 with performances better than or noninferior to the traditional triage method of HPV test and Pap smear, respectively. Comparing to methylated *PCDH10* 24, our markers are more sensitive (63–64% vs. 40%) and less specific (89% vs. 100%) in detecting CIN2 or more severe lesions. Comparing to the data of methylated *CADM1* and *MAL* in a trial of high-risk HPV-infected women 36, the two markers are slightly more sensitive and specific than methylated *MAL* and far more better than methylated *CADM1*. Given that the majority of methylated *PCDHAs* detected in CIN1 were of low intensity in MSP assay, an adoption with quantitative measurement may further improve the specificity of the test. Further head-to-head comparison is required to see which marker is better.

In summary, this study identified collective methylation of clustered *PCDH* genes in cervical cancer. Testing these methylated genes may be valuable in screening or diagnosing cervical neoplasia. Further studies are encouraged to see whether they can also be biomarkers in cancers of other sites.

## Supporting Information

**Table S1.** Primer sequences of *PCDH* genes.

**Table S2.** Performance of methylated PCDHA4 or A13 in combination with HPV16/18 in detection of cervical neoplasia.

**Data S1.** List of hypermethylated genes in CIS and cancer of cervical swab.
