# Supplementary material for: Global methylation silencing of clustered proto-cadherin genes in cervical cancer: serving as diagnostic markers comparable to HPV
Source: Cancer Med. 2014 Nov 21;4(1):43–55. doi: 10.1002/cam4.335 (PMC4312117; doi:10.1002/cam4.335)
Supplement: Supplementary file 1 [file cam40004-0043-sd1.docx]

**Table S1. PCR primers designed for *PCDH* family genes.**

| Genes | | sense primer | antisense primer |
| --- | --- | --- | --- |
| RT-PCR | |  |  |
|  | A4 | GGTACAGTCATCGCCCTGAT | AGCCACCTCCACAGAAACAC |
|  | A8 | GTGTCGGCCTATGAGTTGT | AGCAGCTCTAGCCCTCGTG |
|  | A10 | AACAGCAACAGCTGGACCTT | TCGTGCTGTTCCCTTTCTCT |
|  | A13 | TGCTCACGTTGCTCTGTAT | CAGAACCCAGACAAGGAGGA |
|  | B3 | TGCTCAGGGTGCAGAGAGGGTT | TTGCTACTTGCCTTGAACCAGACCA |
|  | B6 | GTGGATGTGAGCGGCACCGG | CGGGAAGCTATTCCGAGAGGTGGG |
|  | B7 | GCCCTCGTGCGCATTCTGGT | GTGGCGCTGACACTGCCGAT |
|  | B14 | CAGGCATTAAAGGAGCTTCG | ACAATCCCAGCAAAACAAGG |
|  | GB6 | ACCTAGAGCCCCTGGCGGTG | GTGGCAGAGAGTGGCGGCTG |
|  | GB7 | ACAATGCCTGGCTGTCCTAC | CAAGCTATCTGCGAACACCA |
|  | GA12 | CCTATTCCCACGAGGTTTCC | GCCTGAGAGAAACGCCAGT |
|  | GC3 | GCTCACTGTACCGAACACCA | Same as that of GA12 |
| Bisulfite PCR | | |  |
|  | A4BGS | GTAGTTTGGGAGGTAGGGAA | CATCAAATAATTTTTCCAAAAAAAA |
|  | A4MSP | AGGGATATTAACGATAATTCGTCG | ACGCCCTCTAATAAAAACCGA |
|  | A4USP | TGAGGGATATTAATGATAATTTGTTG | AAACACCCTCTAATAAAAACCAAA |
|  | A13BGS | AGGAAGTTATAAAAATTGGGTTTTGA | CCTCCACATAAAAAACCTACAAAAA |
|  | A13MSP | CGTTTTCGAGGAAGTAAAATACGG | GTATCTTTTAAACGCCACCCGAA |
|  | A13USP | TGTTTTTGAGGAAGTAAAATATGG | CATATCTTTTAAACACCACCCAAA |
