# Supplementary material for: Global methylation silencing of clustered proto-cadherin genes in cervical cancer: serving as diagnostic markers comparable to HPV
Source: Cancer Med. 2014 Nov 21;4(1):43–55. doi: 10.1002/cam4.335 (PMC4312117; doi:10.1002/cam4.335)
Supplement: Supplementary file 2 [file cam40004-0043-sd2.doc]

**Table S2. Performance of methylated PCDHA4 or A13 in combination with HPV16/18 in detection of cervical neoplasia.**

| **Test mode** | **Detection target** | **PCDHA4me or HPV16/18** | | | |  | **PCDHA13me or HPV16/18** | | | |  | **PCDHA4me or PCDHA13me or HPV16/18** | | | |
| --- | --- | --- | --- | --- | --- | --- | --- | --- | --- | --- | --- | --- | --- | --- | --- |
|  |  |  |  |  |  |  |  |  |  |  |  |  |  |  |  |
|  |  | Sen % | Spe % | PPV % | NPV % |  | Sen % | Spe % | PPV % | NPV % |  | Sen % | Spe % | PPV % | NPV % |
| Methylated PCDH alone | CIN2+ | 62.6 | 89.0 | 80.2 | 77.0 |  | 64.2 | 89.0 | 80.6 | 77.8 |  | 74.8 | 80.3 | 73.0 | 81.8 |
|  | CIN3+ | 66.3 | 85.9 | 71.9 | 82.5 |  | 71.2 | 87.5 | 75.5 | 84.8 |  | 79.8 | 77.6 | 65.9 | 87.6 |
|  | CA | 84.2 | 75.2 | 33.3 | 97.0 |  | 94.7 | 76.0 | 36.7 | 99.0 |  | 97.4 | 65.5 | 29.4 | 99.4 |
| Methylated PCDH or HPV16/18 | CIN2+ | 76.8 | 79.8 | 73.3 | 82.6 |  | 74.0 | 79.8 | 72.2 | 81.2 |  | 82.1 | 72.3 | 67.8 | 85.0 |
|  | CIN3+ | 81.1 | 76.6 | 65.6 | 88.0 |  | 78.8 | 77.1 | 65.1 | 87.1 |  | 86.5 | 69.3 | 60.4 | 90.5 |
|  | CA | 95.0 | 64.0 | 29.0 | 98.8 |  | 97.4 | 65.5 | 29.4 | 99.4 |  | 97.4 | 56.6 | 24.8 | 99.3 |

Sen: sensitivity, Spe: specificity, PPV: positive predictive value, NPV: negative predictive value.
